# Number of active transcription factor binding sites is essential for the Hes7 oscillator

**DOI:** 10.1186/1742-4682-3-11

**Published:** 2006-02-23

**Authors:** Stefan Zeiser, H Volkmar Liebscher, Hendrik Tiedemann, Isabel Rubio-Aliaga, Gerhard KH Przemeck, Martin Hrabé de Angelis, Gerhard Winkler

**Affiliations:** 1 Institute of Biomathematics and Biometry, GSF-National Research Centre for Environment and Health, Ingolstädter Landstraβe 1, D-85764 Neuherberg, Germany; 2 Department of Mathematics and Computer Science, Ernst-Moritz-Arndt-Universität Greifswald, Jahnstraβe 15a, D-17487 Greifswald, Germany; 3 Institute of Experimental Genetics, GSF-National Research Centre for Environment and Health, Ingolstädter Landstraβe 1, D-85764 Neuherberg, Germany

## Abstract

**Background:**

It is commonly accepted that embryonic segmentation of vertebrates is regulated by a segmentation clock, which is induced by the cycling genes *Hes1 *and *Hes7*. Their products form dimers that bind to the regulatory regions and thereby repress the transcription of their own encoding genes. An increase of the half-life of *Hes7 *protein causes irregular somite formation. This was shown in recent experiments by Hirata et al. In the same work, numerical simulations from a delay differential equations model, originally invented by Lewis, gave additional support. For a longer half-life of the *Hes7 *protein, these simulations exhibited strongly damped oscillations with, after few periods, severely attenuated the amplitudes. In these simulations, the Hill coefficient, a crucial model parameter, was set to 2 indicating that *Hes7 *has only one binding site in its promoter. On the other hand, Bessho et al. established three regulatory elements in the promoter region.

**Results:**

We show that – with the same half life – the delay system is highly sensitive to changes in the Hill coefficient. A small increase changes the qualitative behaviour of the solutions drastically. There is sustained oscillation and hence the model can no longer explain the disruption of the segmentation clock. On the other hand, the Hill coefficient is correlated with the number of active binding sites, and with the way in which dimers bind to them. In this paper, we adopt response functions in order to estimate Hill coefficients for a variable number of active binding sites. It turns out that three active transcription factor binding sites increase the Hill coefficient by at least 20% as compared to one single active site.

**Conclusion:**

Our findings lead to the following crucial dichotomy: either Hirata's model is correct for the *Hes7 *oscillator, in which case at most two binding sites are active in its promoter region; or at least three binding sites are active, in which case Hirata's delay system does not explain the experimental results. Recent experiments by Chen et al. seem to support the former hypothesis, but the discussion is still open.

## Introduction

In mouse embryos, a pair of somites is separated from the anterior end of the presomitic mesoderm every two hours [1]. This process is assumed to be induced by the bHLH factors Hes1 and Hes7 [2,3], which also oscillate with a period of about two hours. Their oscillation is caused by a negative feedback loop in which the proteins repress the transcription of their corresponding genes [4-7]. Hirata et al. [8] showed that the Hes7 protein has a half-life of about 22 minutes. To demonstrate that this is crucial for oscillation, they used mouse mutants with a longer Hes7-half-life of about 30 minutes, but with normal repressor activity. In mice with a smaller protein decay rate, somite segmentation became irregular, and *Hes7 *expression did not show cyclic behaviour.

Lewis [9] used delay differential equations to model the mechanism for the homologous zebrafish *Her1 *and *Her7 *oscillators. Delay equations allow intermediate synthesis steps such as transport, elongation and splicing to be subsumed in the delays. Thus, only two equations are needed, one for the mRNA and one for the protein, in contrast to compartment models where at least three equations are needed. Repression of *Her7 *transcription by Her7 is represented by an inhibitory Hill function. The latter is of sigmoid form and decreases from one to zero. The modulus of steepest descent is called the Hill coefficient. As shown in [10], it correlates with the number of and the cooperativity between transcription factor binding sites. Hirata et al. chose a Hill coefficient of 2, corresponding to a promoter with one single binding site for Hes7 dimers. On the other hand, Bessho et al. [4] showed that Hes7 has one N box and two E boxes as regulatory elements in the promoter region. By transcription analysis they demonstrated that transcription can be repressed by both N box- and E box-containing promoters. Thus, as in *Hes1*, there are at least three binding sites in the regulatory region of *Hes7 *to which Hes7 dimers could bind.

In the present paper, we show that three active transcription-factor binding sites cause an increase of the Hill coefficient, and that such an increase results in a completely different behaviour of the delay system, which does no longer reflects the observations made by Hirata et al. [8].

## Methods

### Model of the Hes7 switch

To compute the Hill coefficient in the *Hes7 *oscillator we use a model recently proposed in [10], which mimics the chemical reactions model for ligand binding in [11,12]. In this approach, the transcriptional activity of the *Hes7 *promoter and its dependence on the concentration of Hes7 is represented by a response function. For convenience, we will approximate the response functions by Hill-type functions later.

**Figure 1:**
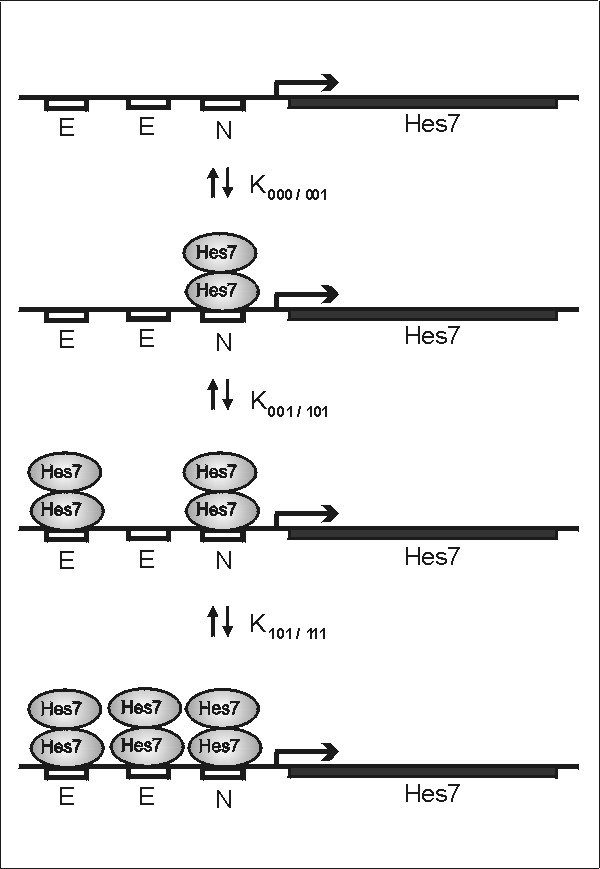
Schematic representation of Hes7-dimer binding in the regulatory region of *Hes7*. Binding sites are indicated by three rectangles. E and N denote an E- or an N-box binding site, respectively. We assume that association and dissociation are in equilibrium. K denotes the respective equilibrium constants. 0 or 1 indicates whether the respective binding sites are occupied or not.

We assume that a single bound dimer represses the transcription of *Hes7 completely*. Then the response function is the long-term relative frequency of occupation of one of the binding sites in dependence on the protein concentration. If [*X*] denotes the Hes7 concentration, the response is given by the ratio of the concentrations [*P*_*U*_] and [*P*_*T*_] of the unoccupied and total promoter configurations:



To express [*P*_*U*_] and [*P*_*T*_] in terms of [*X*], let *ijk *denote a generic promoter configuration. For example, *i *= 1 indicates that the first binding site is occupied and *i *= 0 that it is not; *ijk *= 010 is the configuration where only the second site is occupied. There are six possible reaction channels through which three dimers can bind successively to the three sites (Fig. 1).

We assume that binding of dimers to any promoter configuration is in equilibrium. Let *K*_*ijk*/*hlm *_be the equilibrium constant for the reaction that changes the promoter configuration from *ijk *to *hlm*. Let [*X*_2_] and [*P*_*ijk*_] denote the concentrations of free Hes7 dimers and promoter configurations, respectively. Then we obtain the three equations



We will assume that dimerization is in equilibrium as well. The equilibrium constant of this reaction is *K*_*d *_= [*X*_2_]/[*X*]^2^. For the configuration 000, where no dimer is bound to any of the three binding sites, the equilibrium constants for binding of a dimer to one of the three binding sites are equal, and we may set *K*_*eq *_= *K*_000/*hlm *_for all *h*,*l*,*m*. Under these simplifying assumptions, the response function has the form



see [11]. The constants *γ *and *δ *represent the change in affinity to a dimer of the second and third binding sites. We assume that bound dimers increase the affinity of the remaining unoccupied binding sites, hence *γ*, *δ *≥ 1. In terms of the normalized variable  the response function reads



The steepness of (1) is determined by means of a Hill plot. For this purpose, log *f*_*h*_(*x*)/(1 - *f*_*h*_(*x*)) is plotted against log *x *for 0.1 ≤ *f*_*h*_(*x*) ≤ 0.9. The absolute slope of the regression line for the Hill plot yields a reliable estimate of the Hill coefficient. Then, in the above range, response functions of the form (1) are well approximated by Hill-type functions



with the Hill coefficient *h *and the Hill constant *H*.

### Model of the *Hes7 *oscillator

The temporal course of *Hes7 *mRNA and Hes7 protein concentrations was modelled by delay differential equations. The system reads



where *p*(*t*) and *m*(*t*) denote the amounts of *Hes7 *mRNA and Hes7 proteins at time *t*. The Hill-type function *f*_*h *_in (2) describes the negative feedback of Hes7 protein on *Hes7 *mRNA synthesis. The entries *k *and *a *are the basal transcription rate in the absence of inhibitory proteins, and the rate constant of translation, respectively. Finally, the protein and mRNA decay rates are denoted by *b*and *c*. The latter are inversely proportional to the respective protein and mRNA half-lives *τ*_*p *_and *τ*_*m*_. More precisely, we have *b *= ln2/*τ*_*p *_and *c *= ln2/*τ*_*m*_.

### Numerical simulations

We carried out numerical simulations for the delay system (3) with the different Hill coefficients resulting from the calculations for different binding scenarios sketched above. For numerical integration of the delay system, we used the DDE solver of the software package MATLAB. All parameters except the Hill coefficient were taken from [8]: in particular, the experimentally determined protein half-lives of *τ*_*p *_= 20 min or *τ*_*p *_= 30 min were used as input. The overall delay *T*_*m *_+ *T*_*p *_= 37 min was split into *T*_*m *_= 30 min and *T*_*p *_= 7 min ([8] do not specify *T*_*m *_and *T*_*p*_), which has no influence on the dynamics [13]. The remaining parameters were taken from the original zebrafish model [9]: *Hes7 *mRNA half-life *τ*_*m *_= 3 min, protein synthesis rate *a *= 4.5 molecules per mRNA molecule per min, basal transcription rate *k *= 4.5 mRNA molecules per min, and a Hill constant *H *= 40 protein molecules per cell. The Hill coefficient was varied from 2.0 (the value used in [8]) to 2.4 and 2.6. The latter values were obtained by mathematical analysis of the model for the regulatory region of *Hes7*. Details are reported in the results section.

## Results

### Estimation of the hill coefficient

We calculated the Hill coefficient of the response function (1) for two scenarios.

**(A) **The equilibrium constant *K*_*eq *_of the unoccupied binding sites is not changed by a bound dimer, so *γ *= *δ *= 1. The dimers bind non-synergistically or independently to any one of the three binding sites.

**(B) **A bound dimer changes the equilibrium constant of one of the remaining free binding sites, so the binding is synergistic or (positively) cooperative. Therefore, at least one of the parameters *γ *or *δ *is greater than one.

**Figure 2:**
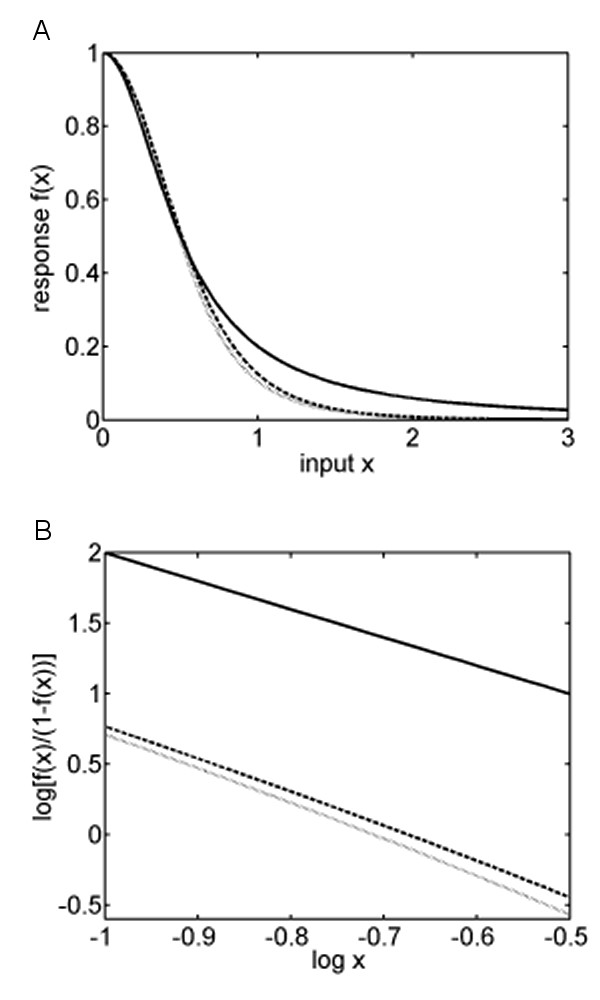
(A) Response functions for a promoter with two (solid line) and three (dashed and dotted lines) binding sites. (B) Hill plots of the three response functions: log(*f*_*h*_(*x*)/(1 - *f*_*h*_(*x*))) is plotted versus log(*x*).

For the case *γ *= *δ *= 1 (A), the response function (1) is plotted as a dashed line in Fig. 2A. If *Hes7 *has only one transcription factor binding site, as assumed by Hirata et al. [8], the response function is a Hill function with a Hill coefficient *h *= 2. For a Hill constant of *H *= 1 it is plotted as a solid line. Fig. 2A shows that an increase in the number of binding sites yields a steeper curve and thus results in increasing strength of the switch. To quantify this, the corresponding Hill plots were constructed (Fig. 2B). For a Hill function with a coefficient of *h *= 2 the Hill plot is a straight line with a slope of -2. The Hill plot of the response function (1) with *γ *= *δ *= 1 is plotted as a dashed line. The slope of the fitted regression line gives a Hill coefficient of about 2.4.

In (B), we assumed synergistic binding of the dimers. As an example, we consider the case where the affinity of the second binding site to Hes7 dimers is increased by 50%, and the affinity of the third binding site is uninfluenced, i.e. *γ *= 1.5, *δ *= 1 (dotted line in Fig 2A). The plot shows that a small increase in the affinity of the second binding site results in a small increase of the strength of the switch. Regression of the Hill plot gives a Hill coefficient equal to 2.6 (Fig. 2B dotted line). Thus, an increase in the number of binding sites or in the affinity of a binding site results in an increase of the Hill coefficient. This effect becomes stronger if the affinity of one of the binding sites is increased by a bound dimer.

### Numerical analysis of the delay system

**Figure 3:**
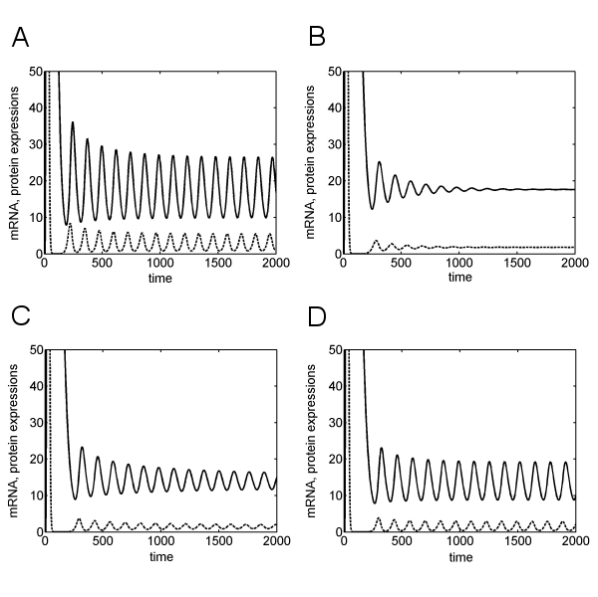
Numerical simulation of the *Hes7 *autoregulatory network for different values for the protein half-life *τ*_*p *_and the Hill coefficient *h*. The expression curves of the mRNA and the protein are given by the dashed and the solid curves, respectively. For better representation, the protein expression curves were scaled by 0.05. (A) *τ*_*p *_= 20 min, *h *= 2. (B) *τ*_*p *_= 30 min, *h *= 2. (C) *τ*_*p *_= 30 min, *h *= 2.4. (D) *τ*_*p *_= 30 min, *h *= 2.6.

We simulated the delay system (3) for the different Hill coefficients calculated above. Figures 3A and 3B display the simulation results for the parameters used in [8]: for a protein half-life of *τ*_*p *_= 20 min and a Hill coefficient of *h *= 2, the system shows undamped oscillations with a period of about 120 min (Fig. 3A). For a greater protein half-life of 30 min, oscillation is strongly damped and the amplitude becomes vanishingly small after four to five cycles (Fig. 3B). This might explain the results found by Hirata and colleagues [8]. There it was shown that cyclic expression of *Hes7 *fails for mouse mutants with a longer Hes7 protein half-life. However, the delay system exhibits a completely different behaviour if the Hill coefficient is increased. For a Hill coefficient equal to 2.4, the damping of the oscillations is much more restrained: After 1700 minutes, during which time more than 14 somites are formed, the oscillation amplitude is greater than after 3 oscillations in the system with a Hill coefficient equal to 2 (Fig. 3C). This effect becomes even stronger when the Hill coefficient is increased further. A Hill coefficient equal to 2.6 leads to a sustained oscillation (Fig. 3D).

## Discussion and conclusion

We used response functions to model the binding of Hes7 dimers to the regulatory region of *Hes7*. Because no experimental data from transcriptional analysis of *Hes7 *were available, we assumed that one bound Hes7 dimer can repress transcription of *Hes7 completely*. We showed that both an increase in the number of binding sites and positive cooperativity increase the value of the Hill coefficient. Taking into account that *Hes7 *has three potential transcription factor binding sites [4], our model suggested an increase of the Hill coefficient of at least 20% compared to a promoter with only one binding site. In the case of independent binding of Hes7 dimers to one of the three binding sites, the Hill coefficient increased from 2 to 2.4. In the case of positive cooperativity, an increase of 50% in the affinity constant of one binding site resulted in a further increase of the Hill coefficient to a value of approximately 2.6.

Numerical analysis of the delay differential equation system proposed by Hirata et al. [8] revealed that oscillations of the *Hes7 *autoregulatory network depend predominantly on the strength of the switch. For a longer half-life of the Hes7 protein, a 20% increase in the Hill coefficient changed the behaviour of the delay system drastically: oscillations become highly damped, and for a Hill coefficient of 2 become insignificant after 5 oscillations. In contrast, a Hill coefficient equal to 2.4 leads only to a weak dampening of the oscillations. After 14 oscillations the system still showed significant amplitudes.

There are two conceivable explanations for these phenomena. On the one hand, if the delay system proposed by Hirata and colleagues [8] describes the *Hes7 *oscillator correctly, their results and our findings suggest a Hill coefficient less than 2.4. If this is the case, there should be no more than two active binding sites in the *Hes7 *promoter. Recent *ex vivo *experiments by Chen et al. [7] support this interpretation. Nevertheless, the following questions are not answered yet:

• There are several potential transcription factor binding sites in the *Hes7 *promoter [4], so why are no more than two of them active?

• Our numerical analysis of the delay system demonstrates that the model is highly sensitive to changes in the Hill coefficient. Is this inherent in the *Hes7 *oscillator or is it just an artefact of the model?

Therefore, it might be helpful to carry out *in vivo *experiments that reveal the underlying mechanisms in the promoter region in more detail. To allow for a more precise estimation of the Hill coefficient, more data will definitely have to be collected.

On the other hand, if further experiments support a higher value of the Hill coefficient, our work shows that the proposed delay system cannot explain irregular somite formation in terms of a longer Hes7 half-life. One possible reason might be that the model is too simple. There might be other mechanisms, hidden in the delay of such a system, that could be influenced by a longer Hes7 protein half-life and explain the effects found by Hirata and colleagues [8]. In this case, a more sophisticated model should be developed.

Let us finally stress once more that further experimental data on the processes in the Hes7 feedback network are required to decide finally on one of the alternatives. For instance, a dose-response curve might be recorded from transcriptional analysis of the Hes7 promoter with various Hes7 dimer concentrations. Then (see the section *Model of the Hes7 switch*) an estimate for the Hill coefficient could be obtained from the Hill plot.
